# Association between triglyceride glucose index and carotid intima‐media thickness in obese and nonobese adults

**DOI:** 10.1111/1753-0407.13312

**Published:** 2022-09-07

**Authors:** Xiaojing Jia, Yuanyue Zhu, Yan Qi, Ruizhi Zheng, Lin Lin, Chunyan Hu, Yi Zhang, Xueyan Wu, Hongyan Qi, Ran Wei, Jie Zhang, Min Xu, Yu Xu, Tiange Wang, Zhiyun Zhao, Yuhong Chen, Yufang Bi, Weiqing Wang, Mian Li, Jieli Lu

**Affiliations:** ^1^ Department of Endocrine and Metabolic Diseases Shanghai Institute of Endocrine and Metabolic Diseases, Ruijin Hospital, Shanghai Jiao Tong University School of Medicine Shanghai China; ^2^ Shanghai National Clinical Research Center for Metabolic Diseases, Key Laboratory for Endocrine and Metabolic Diseases of the National Health Commission of the PR China Shanghai Key Laboratory for Endocrine Tumor, State Key Laboratory of Medical Genomics, Ruijin Hospital, Shanghai Jiao Tong University School of Medicine Shanghai China

**Keywords:** carotid intima‐media thickness, nonlinear relationship, obesity, triglyceride glucose index, 颈动脉内中膜厚度, 非线性关系, 肥胖, 甘油三酯血糖指数

## Abstract

**Background:**

The triglyceride glucose (TyG) index is closely associated with subclinical atherosclerosis. However, the association remains inconclusive among obese and nonobese individuals.

**Methods:**

This prospective study was conducted in 5751 adults with normal carotid intima‐media thickness (CIMT) at baseline. We divided the population into four groups based on the TyG index, which was calculated by the following formula: Ln (fasting triglycerides [mg/dL] × fasting glucose [mg/dL]/2). Information on CIMT was acquired by ultrasonography. Incident elevated CIMT was defined as IMT values greater than 0.9 mm at follow‐up. Odds ratios (ORs) and 95% confidence intervals (CIs) of the associations between TyG index and elevated CIMT were estimated using multivariable logistic regression models.

**Results:**

After a median follow‐up of 4.3 years, 722 (12.6%) individuals had progressed to elevated CIMT. Compared with the second quartile of the TyG index, the first and fourth quartile both conferred higher risks of elevated CIMT after adjusting for potential confounders. In the total population, the ORs for the first and fourth quartile were 1.29 (95% CI, 1.00‐1.66) and 1.42 (95% CI, 1.11‐1.83), respectively. Restricted cubic splines demonstrated an approximately U‐shaped association between TyG index and elevated CIMT among the total and nonobese adults (*P* for nonlinearity <.05), but not in those with general or abdominal obesity.

**Conclusions:**

A U‐shaped association was observed between TyG index and elevated CIMT only among nonobese Chinese adults.

## Graphical abstract

**Highlights**

- Many studies have reported that the triglyceride glucose (TyG) index is an inexpensive biochemical marker to assess metabolic and cardiovascular diseases.
- Our findings indicate that the TyG index is nonlinearly associated with the risk of new‐onset elevated carotid intima‐media thickness (CIMT) during a follow‐up of 4.3 years. We further evaluated such relationship in participants by body size phenotypes. A U‐shaped association between TyG index and elevated CIMT only existed among those without general or abdominal obesity.

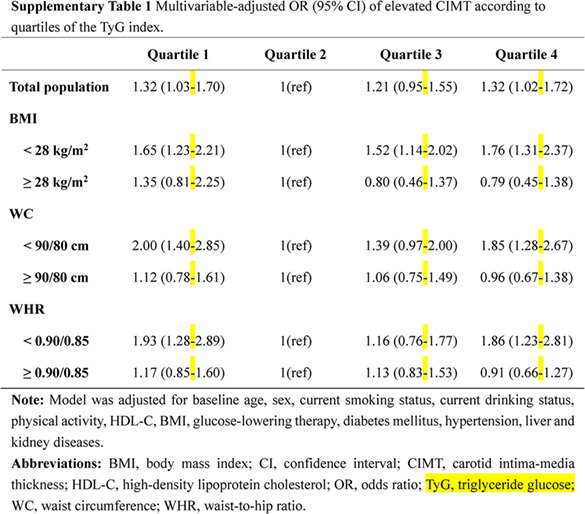

## 摘要

**背景:**

甘油三酯血糖(TyG)指数与亚临床动脉粥样硬化密切相关。然而，肥胖者和非肥胖者之间的联系仍然没有定论。

**方法:**

这项前瞻性研究在5751名基线颈动脉内膜中层厚度(CIMT)正常的成年人中进行。根据TyG指数将人群分为四组，计算公式为:Ln(空腹甘油三酯[mg/dL]×空腹血糖[mg/dL]/2)。超声检查获得CIMT的相关信息。升高的CIMT定义为随访时IMT值大于0.9 mm。用多变量Logistic回归模型估计TyG指数与CIMT升高之间关联的优势比(OR)和95%置信区间(CI)。

**结果:**

经过平均4.3年 的随访，有722例(12.6%)患者进展为CIMT升高。与TyG指数的第二个四分位数相比，在校正了潜在的混杂因素后，第一和第四个四分位数都具有更高的CIMT升高风险。在总人口中，第一和第四个四分位数的OR值分别为1.29(95%CI，1.00~1.66)和1.42(95%CI，1.11~1.83)。限制立方样条法显示TyG指数与非肥胖者和非肥胖者的CIMT升高呈近似的U型相关(P<0.05)，但在一般肥胖者或腹型肥胖者中不存在。

**结论:**

仅在非肥胖的中国成年人中，TyG指数与CIMT升高呈U型相关。

## 1 INTRODUCTION

Cardiovascular diseases (CVDs) remain the major threat to public health and the primary cause of all deaths throughout the world,^1^, ^2^ among which atherosclerotic vascular diseases account for the largest part of health lost.^1^ The carotid intima‐media thickness (CIMT), a surrogate indicator for the presence and progression of atherosclerosis, is associated with future cerebrovascular and cardiovascular events.^3^, ^4^ Assessment of CIMT is now widely used in clinical practice.

Insulin resistance (IR) is an important pathophysiological mechanism of metabolic diseases and CVDs.^5^ The triglyceride glucose (TyG) index is a plausible and inexpensive biochemical marker to assess IR, compared with hyperinsulinemic‐euglycemic clamp (HIEC).^6^ Numerous studies have found that a high TyG index is closely related to type 2 diabetes mellitus,^7^ metabolic syndrome,^8^ and CVD.^9^ However, the risk is debatable at lower levels of TyG index.^10^, ^11^ No significant increase in the risk of CVD is observed with a TyG index lower than 8.45 in the Vascular Metabolic CUN (VMCUN) cohort.^12^ But a J‐shaped relationship of baseline TyG index with the risk of myocardial infarction exists.^10^ In addition, conclusions regarding the correlation between TyG index and the risk of CIMT are still controversial. For example, Zhao et al reported that the TyG index had no significant correlation with carotid hypertrophy (CIMT > 0.9 mm) in the Northern Shanghai Study (NSS),^13^ whereas a higher TyG index was associated with carotid atherosclerosis in patients with ischemic stroke.^14^

Moreover, obesity has a detrimental effect on the cardiovascular system and is strongly linked with CVD.^15^ A previous study found that participants with either overall or central obesity had a higher risk of elevated CIMT among physically inactive participants.^16^ Nevertheless, Hong et al showed that the risks of both incident stroke and myocardial infarction for the highest quartile of the TyG index in the subgroup of body mass index (BMI) < 25 kg/m^2^ were even higher than in the subgroup of BMI ≥ 25 kg/m^2^.^17^ Yet, the relationships between TyG index and CIMT among individuals with different body size phenotypes were not specified.

To address this gap, we investigated the association between TyG index and the risk of elevated CIMT based on a prospective cohort of Chinese community residents. Furthermore, we examined whether the TyG index was a risk factor for elevated CIMT in participants across different body size phenotypes.

## 2 METHODS

### 2.1 Study population

This population‐based study was carried out among adults living in Jiading District, Shanghai, China. The study design has been described elsewhere.^18^, ^19^ Briefly, during February to August 2010, 10 375 men and women aged ≥40 years were enrolled to complete a comprehensive examination of cardiometabolic health. From 2014 to 2015, we invited all eligible participants to come back for a follow‐up visit. For the current analysis, participants diagnosed with CVD (*n* = 850) at baseline were excluded. We further excluded participants who had missing data on CIMT measurements at both baseline (*n* = 74) and follow‐up (*n* = 3618) and elevated CIMT at baseline (*n* = 82). Finally, a total of 5751 subjects were included in the current analysis.

The study conformed to the Institutional Review Board of the Ruijin Hospital, Shanghai Jiaotong University School of Medicine. All participants provided written informed consent.

### 2.2 Data collection and clinical evaluation

At baseline and follow‐up, trained staff from the local community collected data following a standard guideline. The information on sociodemographic characteristics, medical history, and behavioral factors were captured using a comprehensive questionnaire. Current smokers or drinkers were defined according to their daily habits as those who had frequently consumed cigarettes or any alcohol in the past 6 months. Body weight, height, and hip and waist circumference (WC) were measured during visits. WC was measured at the umbilical level in the standing position. BMI was calculated as the weight in kilograms divided by the height in meters squared (kg/m^2^). Waist‐to‐hip ratio (WHR) was computed by dividing the WC by hip circumference. Three systolic and diastolic blood pressure (SBP and DBP) measurements, acquired after ≥5 minutes of rest, were completed by an automated electronic device (OMRON Model HEM‐752 FUZZY; Omron Company, Dalian, China). For the analysis, the average of the three corresponding values was taken. Uniformly trained sonographers with adequate clinical experience used a high‐resolution B‐mode tomographic ultrasound (Esaote Biomedica SpA, Genoa, Italy) to detect and measure both sides of CIMT during two visits. The distance from the first to the second leading edge of the echogenic line was regarded as CIMT of either side. We adopted the value of maximum CIMT in the analysis.

All participants were asked to fast at least 10 hours before taking the 75‐g oral glucose tolerance test. Fasting plasma glucose (FPG) and 2‐hour plasma glucose concentrations were quantified by applying an autoanalyzer (Modular P800; Roche, Basel, Switzerland) and the glucose oxidase or hexokinase method at local hospitals. The level of glycated hemoglobin (HbA1c) was measured by using the method of high‐performance liquid chromatography (D‐10; Bio‐Rad, Hercules, California). Fasting levels of triglycerides (TGs), total cholesterol (TC), low‐density lipoprotein cholesterol (LDL‐C), and high‐density lipoprotein cholesterol (HDL‐C) were also analyzed by an automated analyzer (Modular Analytics P800 and Modular E170; Roche, Basel, Switzerland).

### 2.3 Definitions and diagnostic criteria

Participants with a BMI ≥ 28.0 kg/m^2^ were defined as generally obese according to the characteristics of the Chinese population.^20^ Abdominal obesity was considered as a WC ≥ 90 cm or a WHR > 0.90 for men and a WC ≥ 80 cm or a WHR > 0.85 for women.^21^, ^22^ The TyG index was calculated as Ln (fasting TGs [mg/dL] × fasting glucose [mg/dL]/2).^23^ Individuals with a CIMT greater than 0.9 mm at follow‐up but a low CIMT at baseline were considered to have new‐onset elevated CIMT.^24^ Diabetes was defined as FPG ≥ 126 mg/dL (7.0 mmol/L), or 2‐hour plasma glucose ≥200 mg/dL (11.1 mmol/L), or HbA1c ≥ 6.5% (48 mmol/mol), or a self‐reported previous diagnosis of diabetes by physicians and using insulin or taking antidiabetic medications.^25^ Hypertension was defined as SBP ≥ 140 mm Hg, DBP ≥ 90 mm Hg, the use of antihypertensive medication or previous diagnosis of hypertension by health‐care professionals. The history of diagnosed liver (eg, viral or autoimmune hepatitis, cirrhosis, liver cancer, or other) and kidney (eg, kidney stone, glomerulonephritis, nephropathy, or other) diseases was self‐reported.

### 2.4 Statistical analysis

Baseline characteristics were presented by the quartiles of the TyG index. Continuous variables were expressed in means (standard deviations). Categorical variables were expressed as numbers (percentages). To compare the differences of baseline characteristics between groups, we conducted one‐way analyses of variance and chi‐square tests. In the current study, we used the TyG index at baseline (2010) to predict the subsequent risk of new‐onset elevated CIMT. The odds ratios (ORs) and 95% confidence intervals (CIs) were calculated by a logistic regression model and adjusted for baseline age, sex, current smoking status, current drinking status, physical activity, HDL‐C, BMI, and glucose‐lowering therapy.

Stratified analyses were further performed to evaluate the above‐mentioned relationships by BMI (<28 and ≥28 kg/m^2^), WC (<90/80 and ≥90/80 cm for males and females, respectively), and WHR (<0.90/0.85 and ≥0.90/0.85 for males and females, respectively). All tests were two sided, and a *P* value <.05 was considered statistically significant. All of the above statistical analyses were performed using SAS version 9.4 (SAS Institute, Cary, North Carolina).

We also used R version 4.0.5 (R Foundation for Statistical Computing, Vienna, Austria) to construct restricted cubic spline models with four knots to detect whether there was a nonlinear relationship between TyG index and the risk of elevated CIMT after full adjustment.

## 3 RESULTS

Baseline characteristics of the 5751 participants are displayed by TyG index quartiles in Table 1. The mean TyG index quartiles were 8.02 ± 0.22, 8.48 ± 0.11, 8.86 ± 0.12, and 9.54 ± 0.45, respectively. As expected, SBP, DBP, TGs, FPG, and CIMT were more likely to increase across the quartiles. There was no significant difference in the lifestyle factors (smoking and drinking) and education level among the four groups.

**TABLE 1:** Baseline characteristics of participants according to quartiles of TyG index

Characteristic	Quartile 1 (*n* = 1438)	Quartile 2 (*n* = 1437)	Quartile 3 (*n* = 1439)	Quartile 4 (*n* = 1437)	*P*
Age (y)	56.2 ± 9.1	57.6 ± 8.6	57.9 ± 8.3	58.0 ± 8.2	<.001
Male (*n*, %)	506 (24.2)	521 (24.9)	526 (25.1)	541 (25.8)	.592
BMI (kg/m^2^)	23.8 ± 3.0	24.8 ± 3.0	25.7 ± 3.1	26.4 ± 3.0	<.001
Waist circumference (cm)	77.91 ± 8.22	81.48 ± 8.07	83.81 ± 8.38	86.33 ± 8.04	<.001
Hip circumference (cm)	92.09 ± 5.58	93.58 ± 5.46	94.72 ± 5.63	95.38 ± 5.61	<.001
Waist‐to‐hip ratio	0.85 ± 0.06	0.87 ± 0.06	0.88 ± 0.06	0.91 ± 0.05	<.001
Current smoker (*n*, %)	279 (20.6)	274 (20.2)	289 (21.5)	309 (22.7)	.400
Current drinker (*n*, %)	147 (10.9)	136 (10.0)	129 (9.5)	162 (11.9)	.189
High school education or above (*n*, %)	302 (21.0)	285 (19.8)	271 (18.8)	265 (18.4)	.451
Glucose‐lowering therapy (*n*, %)	18 (1.3)	45 (3.1)	111 (7.7)	214 (14.9)	<.001
SBP (mm Hg)	134.8 ± 19.2	139.6 ± 19.2	142.5 ± 19.4	146.9 ± 19.1	<.001
DBP (mm Hg)	79.9 ± 10.1	82.6 ± 9.9	83.7 ± 10.0	86.2 ± 10.1	<.001
TC (mg/dL)	189.26 ± 31.54	203.35 ± 34.97	210.91 ± 35.69	222.25 ± 44.75	<.001
LDL‐C (mg/dL)	108.72 ± 26.05	124.13 ± 29.78	131.47 ± 31.15	128.66 ± 39.06	<.001
HDL‐C (mg/dL)	58.18 ± 12.45	53.02 ± 11.72	49.37 ± 10.77	44.82 ± 10.27	<.001
TGs (mg/dL)	70.53 ± 14.57	105.81 ± 16.19	146.06 ± 26.02	276.54 ± 185.08	<.001
FPG (mg/dL)	88.46 ± 10.08	93.27 ± 12.51	99.24 ± 18.51	117.13 ± 41.35	<.001
PBG (mg/dL)	113.64 ± 37.65	126.31 ± 48.11	148.81 ± 66.93	195.66 ± 103.98	<.001
TyG index	8.02 ± 0.22	8.48 ± 0.11	8.86 ± 0.12	9.54 ± 0.45	<.001
CIMT (mm)	0.56 ± 0.09	0.57 ± 0.09	0.58 ± 0.09	0.58 ± 0.09	<.001

*Note*: Data are presented as means (standard deviations) or medians (interquartile ranges) for continuous variables or numbers (percentages) for categorical variables.

Abbreviations: BMI, body mass index; CIMT, carotid intima‐media thickness; DBP, diastolic blood pressure; FPG, fasting plasma glucose; HDL‐C, high‐density lipoprotein cholesterol; LDL‐C, low‐density lipoprotein cholesterol; PBG, postprandial blood glucose; SBP, systolic blood pressure; TC, total cholesterol; TGs, triglycerides; TyG, triglyceride glucose.

After a median follow‐up of 4.3 years, 165 (11.5%), 152 (10.6%), 193 (13.4%), and 212 (14.8%) participants had elevated CIMT in four quartiles of the TyG index, respectively. As presented in Table 2, we explored the associations by quartiles of the TyG index, with the second quartile serving as the reference. After adjusting for potential confounding variables (model 3), the ORs of elevated CIMT were 1.29 (95% CI, 1.00‐1.66), 1.24 (95% CI, 0.97‐1.59), and 1.42 (95% CI, 1.11‐1.83) for the first, third, and fourth TyG index quartile, respectively. Similar results were observed when we further adjusted for diabetes mellitus, hypertension, and liver and kidney diseases (Table S1). These results indicate a nonlinear association between TyG index and elevated CIMT.

**TABLE 2:** Odds ratios (95% CI) for risk of elevated CIMT according to quartiles of TyG index

	Quartile 1	Quartile 2	Quartile 3	Quartile 4
Cases (incidence, %)	165 (11.5)	152 (10.6)	193 (13.4)	212 (14.8)
Model 1	1.10 (0.87‐1.38)	1 (ref)	**1.31 (1.04‐1.64)**	**1.46 (1.17‐1.83)**
Model 2	1.19 (0.94‐1.51)	1 (ref)	**1.31 (1.04‐1.64)**	**1.46 (1.16‐1.83)**
Model 3	**1.29 (1.00‐1.66)**	1 (ref)	1.24 (0.97‐1.59)	**1.42 (1.11‐1.83)**

*Note*: Model 1 was an unadjusted model. Model 2 was adjusted for baseline age. Model 3 was adjusted for baseline age, sex, current smoking status, current drinking status, physical activity, HDL‐C, BMI, and glucose‐lowering therapy. The bold values indicated statistical significance.

Abbreviations: BMI, body mass index; CIMT, carotid intima‐media thickness; HDL‐C, high‐density lipoprotein cholesterol; TyG, triglyceride glucose.

Subgroup analyses for obese and nonobese participants are shown in Table 3. The association between TyG index and CIMT was only significant in those without general or abdominal obesity. In the multivariable model, the second quartile of the TyG index had the lowest risk of elevated CIMT among participants with BMI < 28 kg/m^2^. Compared to the second quartile, the risk increased significantly in both quartile 1 (OR 1.96; 95% CI, 1.37‐2.79) and quartile 4 (OR 1.95; 95% CI, 1.36‐2.79) of the TyG index in individuals with normal WC (<90 cm in men and <80 cm in women). Similarly, the fully adjusted ORs (95% CI) for elevated CIMT among participants with normal WHR (<0.90 in men and <0.85 in women) were 1.91 (1.27‐2.86) and 1.92 (1.28‐2.90) in quartile 1 and quartile 4, respectively. The risk estimates did not change significantly in sensitivity analysis with further adjustments for diabetes mellitus, hypertension, and liver and kidney diseases (Table S1), whereas there was no relationship between TyG index and elevated CIMT in obese participants.

**TABLE 3:** Subgroup analyses for risk of elevated CIMT by TyG index

	Quartile 1	Quartile 2	Quartile 3	Quartile 4
BMI				
<28 kg/m^2^				
Model 1	**1.35 (1.03‐1.77)**	1 (ref)	**1.59 (1.22‐2.07)**	**1.80 (1.39‐2.33)**
Model 2	**1.46 (1.11‐1.93)**	1 (ref)	**1.54 (1.18‐2.01)**	**1.75 (1.35‐2.27)**
Model 3	**1.60 (1.20‐2.15)**	1 (ref)	**1.56 (1.17‐2.07)**	**1.89 (1.41‐2.52)**
≥28 kg/m^2^				
Model 1	1.17 (0.72‐1.90)	1 (ref)	1.00 (0.61‐1.65)	1.04 (0.63‐1.70)
Model 2	1.15 (0.70‐1.88)	1 (ref)	0.96 (0.58‐1.60)	1.07 (0.65‐1.76)
Model 3	1.32 (0.79‐2.19)	1 (ref)	0.85 (0.50‐1.45)	0.87 (0.51‐1.48)
WC				
<90/80 cm				
Model 1	**1.56 (1.13‐2.16)**	1 (ref)	**1.42 (1.02‐1.97)**	**1.80 (1.31‐2.47)**
Model 2	**1.82 (1.30‐2.54)**	1 (ref)	**1.43 (1.02‐2.00)**	**1.90 (1.37‐2.64)**
Model 3	**1.96 (1.37‐2.79)**	1 (ref)	1.41 (0.99‐2.02)	**1.95 (1.36‐2.79)**
≥90/80 cm				
Model 1	0.90 (0.64‐1.26)	1 (ref)	1.23 (0.90‐1.69)	1.19 (0.86‐1.63)
Model 2	0.95 (0.67‐1.33)	1 (ref)	1.21 (0.87‐1.66)	1.20 (0.87‐1.66)
Model 3	1.08 (0.75‐1.54)	1 (ref)	1.10 (0.78‐1.55)	1.05 (0.74‐1.50)
WHR				
<0.90/0.85				
Model 1	**1.49 (1.03‐2.16)**	1 (ref)	1.14 (0.78‐1.69)	**1.74 (1.22‐2.50)**
Model 2	**1.75 (1.19‐2.57)**	1 (ref)	1.18 (0.79‐1.76)	**1.78 (1.22‐2.58)**
Model 3	**1.91 (1.27‐2.86)**	1 (ref)	1.19 (0.78‐1.81)	**1.92 (1.28‐2.90)**
≥0.90/0.85				
Model 1	0.98 (0.73‐1.31)	1 (ref)	1.27 (0.96‐1.68)	1.08 (0.81‐1.44)
Model 2	0.99 (0.73‐1.32)	1 (ref)	1.27 (0.96‐1.68)	1.11 (0.83‐1.48)
Model 3	1.11 (0.81‐1.52)	1 (ref)	1.19 (0.88‐1.60)	1.04 (0.76‐1.43)

*Note*: Obesity was defined as a BMI of 28.0 kg/m^2^ or higher. Abdominal obesity was defined as WC ≥ 90 cm or WHR > 0.90 in men and WC ≥ 80 cm or WHR > 0.85 in women. Model 1 was an unadjusted model. Model 2 was adjusted for baseline age. Model 3 was adjusted for baseline age, sex, current smoking status, current drinking status, physical activity, HDL‐C, BMI, and glucose‐lowering therapy. The bold values indicated statistical significance.

Abbreviations: BMI, body mass index; CIMT, carotid intima‐media thickness; HDL‐C, high‐density lipoprotein cholesterol; TyG, triglyceride glucose; WC, waist circumference; WHR, waist‐to‐hip ratio.

**FIGURE 1:**
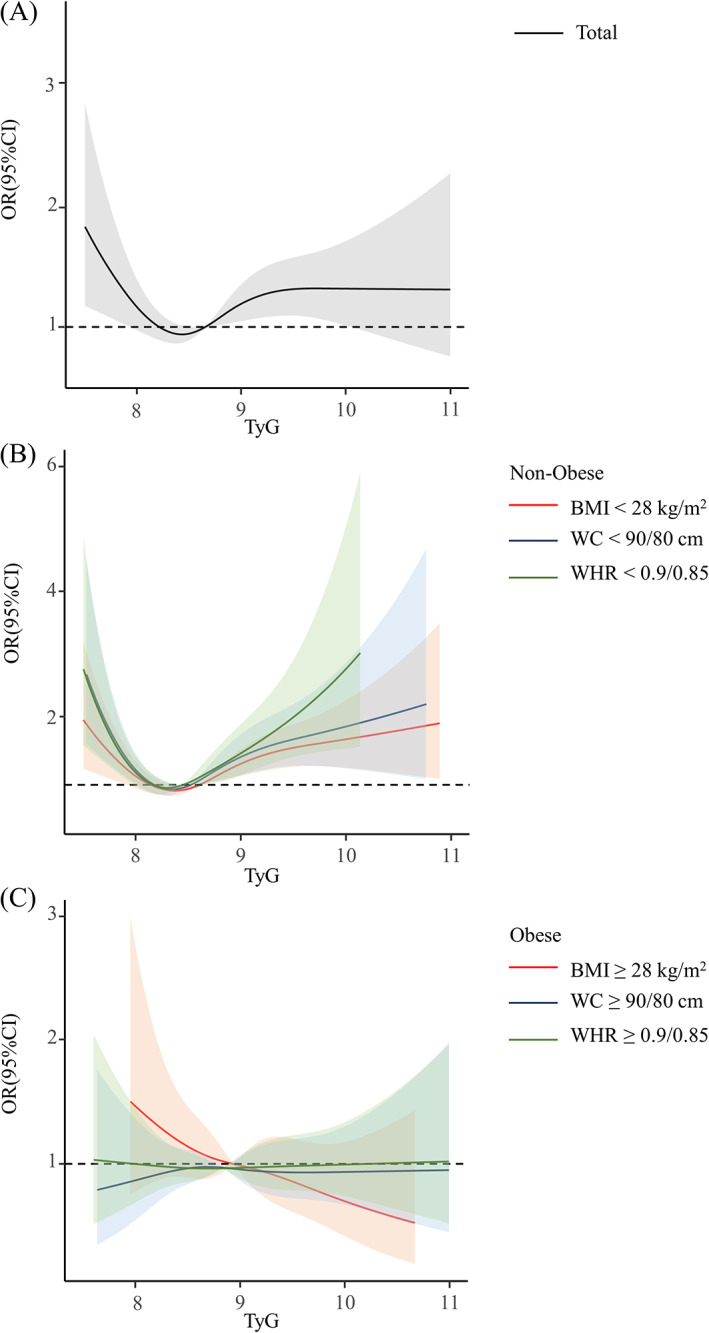
**The association between baseline TyG level and incident elevated CIMT based on restricted cubic splines.** The solid lines represent a fitted relationship and the shadows represent the 95% confidence interval among (A) total, (B) non‐obese and (C) obese population. Model was adjusted for baseline age, sex, current smoking status, current drinking status, physical activity, HDL‐C, BMI and glucose‐lowering therapy.

Restricted cubic splines were additionally applied to model and display the association between TyG index and elevated CIMT, with confounders adjusted (Figure 1). There was an approximately U‐shaped association among the total and nonobese population. As presented in Figure 1A, the risk of elevated CIMT reached a nadir when TyG indices were within the interval of 7.95 to 8.66 in the total population. The association was negative below these values, but positive above (*P* for nonlinearity = .0039). In nonobese participants (Figure 1B), a marked increase in risk was observed at both low and high levels of the TyG index. Among individuals with BMI < 28 kg/m^2^, we noticed that the risk of elevated CIMT was significantly reduced at TyG indices in the range of 7.91 to 8.60 (*P* for nonlinearity = .0003). Also, a strong nonlinear relationship existed in participants with WC < 90/80 cm or WHR < 0.90/0.85 (*P* for nonlinearity < .0001). The risk touched the bottom when the TyG index was around 8.33 and 8.31, respectively. However, no apparent association was found in the subgroups of BMI ≥ 28 kg/m^2^, WC ≥ 90/80 cm, and WHR ≥ 0.90/0.85 (*P* for nonlinearity = .9475, .8846 and .9707, respectively) (Figure 1C).

## 4 DISCUSSION

In the current study, we found a nonlinear association between the baseline TyG index and risk of new‐onset elevated CIMT. Moreover, the relationship was only significant among those without general or abdominal obesity. Our finding provides evidence that the TyG index is useful in identifying those at high risk of subclinical atherosclerosis, especially among nonobese individuals.

Substantial studies have reported that a high baseline TyG index is associated with elevated CIMT. Recently, a study of 631 subjects demonstrated that subclinical atherosclerosis, defined as CIMT > 0.9 mm, correlated with the TyG index.^26^ In addition, a prospective study based on the Beijing Health Management Cohort (BHMC) and the Beijing Physical Examination Cohort (BPEC) indicated that the highest quartile of the TyG index increased the risk of carotid plaque compared with those in the lowest quartile.^27^ In accordance with these studies, we also find that a higher TyG index is associated with higher risk of elevated CIMT. In addition, our finding indicates that the relationship is mostly significant in nonobese individuals.

The TyG index is a surrogate indicator of IR, which is generally recognized as the driving force of CVD events. Endothelial cells (ECs) are the other target tissue linked to IR, as are liver and skeletal muscle.^28^ Increasing oxidative stress in ECs with significant contributions from NADPH oxidase, mitochondria, and uncoupled eNOS results in decreasing availability of NO.^29^, ^30^ Impaired endothelium‐derived NO might fail to inhibit both leukocyte adhesion and related promoters,^31^ driving atherosclerotic development.

Moreover, the current analysis shows that the TyG index is U‐shaped associated with the risk of elevated CIMT, and those in the second quartile have the lowest risk. Numerous publications indicate that there is a nonlinear relationship between TyG index and cardiovascular mortality.^32^ Meanwhile, among the middle‐aged and elderly US population in the National Health and Nutrition Examination Survey (NHANES), the second quartile of the TyG index encompasses the lowest risk of cardiovascular mortality (hazard ratio 0.62; 95% CI, 0.43‐0.88) when compared with the first quartile.^33^ Lower TyG index also demonstrates higher risk.

We notice that participants in the first quartile of the TyG index with lower BMI, TGs, and FPG at baseline have higher risk of CIMT progression, compared with those in the second quartile of the TyG index. Several potential mechanisms underlying this association have been postulated. Very low levels of TGs or FPG, approximately equal to a low TyG index, may produce an adverse effect on the occurrence of CVD. It was reported previously that FPG was nonlinearly related to CVD, and lower FPG was slightly associated with increased risk of stroke and atherosclerotic CVD.^34^, ^35^ Animal experiments demonstrate that repeated hypoglycemia enhances neointima formation after vascular injury,^36^ indicating that lower blood glucose predisposes to elevated CIMT. Moreover, low TG levels may play a negative role on the vascular endothelium. For example, low cholesterol levels could increase the permeability of erythrocyte membranes.^37^ As determined by the two parameters, a lower TyG index may show an increased risk of elevated CIMT.

Given that adiposity substantially affects insulin sensitivity,^38^ different levels of adiposity might influence the status of IR.^39^ To explore whether there is an association between TyG index and elevated CIMT, we introduced subgroup analyses. The current study shows that higher levels of TyG index are significantly associated with the risk of new‐onset elevated CIMT, but only in those without general or abdominal obesity. In other words, the TyG index may have a better predictive value in nonobese individuals. Similarly, a recent cross‐sectional study of 473 postmenopausal women stratified by BMI has shown that there is an increased risk of subclinical atherosclerosis with TyG index only in lean women.^40^

However, the association which is mainly significant in nonobese individuals has not been elucidated. Nevertheless, there are several speculations. First, the TyG index is a predictor of metabolic dysregulation and may better reflect the risk of metabolic diseases in nonobese individuals.^41^ But obese individuals, who typically have several metabolic risk factors,^42^ have a greater burden than lean participants, which would obscure the predictive role of the TyG index in elevated CIMT. Moreover, the TyG index may be more reflective of muscle‐related IR and correlates strongly with homeostasis model assessment of insulin resistance (HOMA‐IR) in healthy nonobese individuals,^43^, ^44^ as increased intramyocellular TG accumulation would impair glucose uptake and insulin signaling in muscle.^45^, ^46^ On the other hand, obese people have strong liver^47^ rather than muscle IR. Finally, inherited genetic factors could partly explain that the TyG index is significant in nonobese individuals. A two‐stage genome‐wide association meta‐analysis identified a locus near *insulin receptor substrate 1 (IRS1)* gene that is associated with reduced body fat percentage but also with an impaired metabolic profile, including increased IR and risk of coronary artery disease.^48^

Previous studies have pointed out that age is strongly and linearly associated with CIMT.^49^ An increase in CIMT with age can be expected. As shown in Table S2, CIMT increased during follow‐up, while the risk attributed to increasing age may be similar across the four groups.^49^ Despite the fact that age has an inevitable effect on CIMT progression, the TyG index itself even works after adjustment for baseline age (Table 2).

The main strengths of this study include the prospective design, large sample size, and comprehensive assessment of covariates. In addition, the results of combined subgroup analyses can provide further information on the predictive value for certain groups. Nevertheless, we acknowledge that several limitations exist in our study. First, the statistical power might be slightly compromised due to the shorter follow‐up. Second, the assessment of CIMT and TyG index is conducted at baseline and follow‐up, ignoring the dynamic changes of the TyG index. Long‐term investigations might address this issue. Third, although many studies have reported that CIMT measurements are useful in the evaluation of CVD risk, the effectiveness is still uncertain. Fourth, the findings could not be extended to younger populations, given our study is consisted of a Chinese population over 40 years.

In conclusion, we report that the baseline TyG index is nonlinearly associated with the risk of elevated CIMT in middle‐aged and elderly nonobese Chinese adults, suggesting that this simple index might be useful in preventing subclinical atherosclerosis.

## AUTHOR CONTRIBUTIONS

Xiaojing Jia, Yuanyue Zhu, Weiqing Wang, Mian Li, and Jieli Lu contributed to the study design and concept. Xiaojing Jia, Yuanyue Zhu, and Yan Qi analyzed the data and drafted the manuscript. Ruizhi Zheng, Lin Lin, Chunyan Hu, Yi Zhang, Xueyan Wu, Hongyan Qi, Ran Wei, and Jie Zhang contributed to data interpretation and editing of the manuscript. Min Xu, Yu Xu, Tiange Wang, Zhiyun Zhao, Yuhong Chen, and Yufang Bi critically revised the manuscript for important intellectual content. All authors were involved in writing and revising the paper and gave their final approval of the submitted and published versions. Weiqing Wang, Mian Li, and Jieli Lu guarantee this work, have full access to the data, and take responsibility for the integrity of the data.

## FUNDING INFORMATION

This study was supported by grants from the National Natural Science Foundation of China (81870604, 81970691, 91857205, 82088102, 81930021, 82170819), the Shanghai Medical and Health Development Foundation (Grant No. DMRFP_I_01), the Shanghai Outstanding Academic Leaders Plan (Grant No. 20XD1422800), the Clinical Research Plan of SHDC (Grant No. SHDC2020CR3064B), and the Shanghai Science and Technology Committee (Grant No. 20Y11905100).

## CONFLICT OF INTEREST

The authors declare that they have no conflict of interest.

## Supporting information


**APPENDIX S1** Supporting information
