# Supplementary material for: Association between triglyceride glucose index and carotid intima‐media thickness in obese and nonobese adults
Source: J Diabetes. 2022 Sep 7;14(9):596–605. doi: 10.1111/1753-0407.13312 (PMC9512765; doi:10.1111/1753-0407.13312)
Supplement: Supplementary file 1 — APPENDIX S1 Supporting information [file JDB-14-596-s001.docx]

**Supplementary materials:**

**Supplementary Table 1** Multivariable-adjusted OR (95% CI) of elevated CIMT according to quartiles of the TyG index.

|  | Quartile 1 | Quartile 2 | Quartile 3 | Quartile 4 |
| --- | --- | --- | --- | --- |
| Total population | 1.32 (1.03-1.70) | 1(ref) | 1.21 (0.95-1.55) | 1.32 (1.02-1.72) |
| BMI |  |  |  |  |
| < 28 kg/m^2^ | 1.65 (1.23-2.21) | 1(ref) | 1.52 (1.14-2.02) | 1.76 (1.31-2.37) |
| ≥ 28 kg/m^2^ | 1.35 (0.81-2.25) | 1(ref) | 0.80 (0.46-1.37) | 0.79 (0.45-1.38) |
| WC |  |  |  |  |
| < 90/80 cm | 2.00 (1.40-2.85) | 1(ref) | 1.39 (0.97-2.00) | 1.85 (1.28-2.67) |
| ≥ 90/80 cm | 1.12 (0.78-1.61) | 1(ref) | 1.06 (0.75-1.49) | 0.96 (0.67-1.38) |
| WHR |  |  |  |  |
| < 0.90/0.85 | 1.93 (1.28-2.89) | 1(ref) | 1.16 (0.76-1.77) | 1.86 (1.23-2.81) |
| ≥ 0.90/0.85 | 1.17 (0.85-1.60) | 1(ref) | 1.13 (0.83-1.53) | 0.91 (0.66-1.27) |

**Note:** Model was adjusted for baseline age, sex, current smoking status, current drinking status, physical activity, HDL-C, BMI, glucose-lowering therapy, diabetes mellitus, hypertension, liver and kidney diseases.

**Abbreviations:** BMI, body mass index; CI, confidence interval; CIMT, carotid intima-media thickness; HDL-C, high-density lipoprotein cholesterol; OR, odds ratio; TyG, triglyceride glucose; WC, waist circumference; WHR, waist-to-hip ratio.

**Supplementary Table 2** Characteristics of participants according to the quartiles of baseline TyG index at follow-up.

| Follow-up characteristics | Quartile1 (n=1438) | Quartile 2 (n=1437) | Quartile 3 (n=1439) | Quartile 4 (n=1437) | *p* |
| --- | --- | --- | --- | --- | --- |
| Age (years) | 60.4 ± 9.0 | 61.8 ± 8.5 | 62.1 ± 8.3 | 62.3 ± 8.2 | <0.001 |
| BMI (kg/m^2^) | 23.7 ± 3.9 | 24.6 ± 3.3 | 25.4 ± 5.4 | 26.1 ± 7.2 | <0.001 |
| Waist circumference (cm) | 79.77 ± 9.77 | 82.82 ± 10.01 | 84.72 ± 9.82 | 86.34 ± 9.99 | <0.001 |
| Hip circumference (cm) | 92.24 ± 8.40 | 93.63 ± 7.51 | 94.61 ± 7.51 | 94.84 ± 7.17 | <0.001 |
| Waist-to-hip ratio | 0.87 ± 0.12 | 0.89 ± 0.12 | 0.90 ± 0.12 | 0.91 ± 0.09 | <0.001 |
| Glucose-lowering therapy (n, %) | 24 (1.7) | 59 (4.1) | 156(10.8) | 324 (22.6) | <0.001 |
| SBP (mmHg) | 129.9 ± 16.8 | 133.5 ± 16.8 | 135.6 ± 16.8 | 138.8 ± 17.1 | <0.001 |
| DBP (mmHg) | 75.0 ± 9.5 | 76.4 ± 9.5 | 76.6 ± 9.3 | 77.7 ± 9.7 | <0.001 |
| TC (mg/dL) | 192.58 ± 33.03 | 201.84 ± 36.73 | 206.48 ± 37.13 | 211.51 ± 43.45 | <0.001 |
| LDL-C (mg/dL) | 128.82 ± 26.07 | 138.61 ± 29.00 | 142.94 ± 29.42 | 145.51 ± 32.48 | <0.001 |
| HDL-C (mg/dL) | 56.57 ± 12.06 | 52.56 ± 11.61 | 50.17 ± 10.50 | 46.90 ± 9.91 | <0.001 |
| TGs (mg/dL) | 99.09 ± 39.94 | 134.97 ± 64.62 | 168.36 ± 88.28 | 245.80 ± 160.25 | <0.001 |
| FPG (mg/dL) | 100.55 ± 13.79 | 105.44 ± 16.66 | 111.09 ± 24.02 | 124.08 ± 36.12 | <0.001 |
| PBG (mg/dL) | 130.55 ± 46.76 | 147.54 ± 54.02 | 165.94 ± 68.30 | 195.52 ± 83.36 | <0.001 |
| TyG | 8.44 ± 0.38 | 8.78 ± 0.39 | 9.03 ± 0.45 | 9.45 ± 0.57 | <0.001 |
| CIMT (mm) | 0.70 ± 0.16 | 0.70 ± 0.14 | 0.71 ± 0.15 | 0.71 ± 0.16 | 0.004 |

**Note:** Data were presented as means (standard deviations) or medians (interquartile ranges) for continuous variables, or numbers (percentages) for categorical variables.

**Abbreviations:** BMI, body mass index; CIMT, carotid intima-media thickness; DBP, diastolic blood pressure; FPG, fasting plasma glucose; HDL-C, high-density lipoprotein cholesterol; LDL-C, low-density lipoprotein cholesterol; PBG, postprandial blood glucose; SBP, systolic blood pressure; TC, total cholesterol; TGs, triglycerides; TyG, triglyceride glucose.
